# A Personal Perspective: Is Bullying Still a Problem in Medicine?

**DOI:** 10.2147/AMEP.S297835

**Published:** 2021-02-10

**Authors:** Simon D Taylor-Robinson, Paulo A De Sousa Lopes, Jey Zdravkov, Rachel Harrison

**Affiliations:** 1 Department of Surgery and Cancer, Imperial College London, London, UK; 2 Department of Medicine, UAI Universidad Abierta Interamericana, Buenos Aires, Argentina; 3 Dean Street Sexual Health Clinic, London, UK; 4 Department of South East Asian Studies, School of Oriental and African Studies, London, UK

**Keywords:** bullying, institutional bullying, personal bullying, unconscious bias

## Abstract

Bullying of whatever form should have no place in the Medical Profession. Reforms to junior doctor training and reduction in working hours have helped to control most of the individual bullying which may have existed in the past. However, the complexities of institutional bullying still exist. In the United Kingdom, centralised monitoring systems, such as Athena SWAN, are designed to reward academic and medical institutions for positive steps to introduce equality and mitigate bullying. However, the reality is that such processes may be conducted in healthcare or educational establishments that have little intention to address the problem thoroughly. We report the personal experience of both individual and institutional bullying in the medical career of a medically-qualified interviewee and reflect on ways to mitigate the problem. We also consider whether unconscious bias affects our relationships with patients. In a caring medical profession, there should be no room for intolerance, unconscious bias or bullying.

## Introduction

Over the course of time, we have had recourse on many an occasion to consider what effect a seemingly vocational profession has had on those who practice it. Stress, burn-out, failed relationships, divorce and even suicide are some of the often talked about aspects of a career which can be all-consuming for many.^1^

However, there has always been a group of people who have attempted to alleviate the seeming tyranny of a medical career by “taking it out” on others. We think, over the years, the way that this has been exacted has changed to being more organisational and institutional, rather than individual, but nevertheless, the problem still exists with undertones or even overtones of structural racism, gender and disability representational imbalance, anti-Semitism, Islamophobia, trans- or homophobia.

We report the experience of a well-known medically-qualified practitioner who spoke to the authors about his experiences of personal and institutional bullying in a medical career spanning more than 40 years. He felt that his experiences were typical of those experienced by his generation of medical practitioners in the United Kingdom during the period from the early 1980s.

## Methods

This personal perspective on personal and institutional bullying in the British Medical profession was drawn from direct discussions and the daily diary of a British medically-qualified individual in the period from August 1984 to October 2019. The individual (termed the “reportee”) was known to all four authors. The reportee took the initiative and approached all four authors to write his personal perspective of bullying in a narrative and non-scientific way, in order to discuss what the reportee felt could be ongoing issues in medical institutions in the United Kingdom. In addition to making the bullying aspects of his diaries available (all of which are reported in a non-selective way), the reportee agreed to take part in interviews with the four authors in order to highlight the fact that he felt that bullying is a little discussed, but unresolved problem in British Medicine. We report the content of the interviews in a non-selective way and have not excluded anything that was discussed. The local Research Ethics Committee did not require ethical approval for the authors to speak to the reportee, provided written, informed consent was obtained. Accordingly, written, informed consent was obtained from the reportee for publication of the diary extracts and for transcription of the conversations, prior to any discussions being had with any of the authors. The reportee read the final draft of the manuscript and was happy with its content for publication. He was happy that he could not be identified through descriptions of the incidents discussed. All those mentioned in the acknowledgement section read the manuscript and gave their permission to be acknowledged for their help and guidance in the generation of this manuscript.

## Results

### Early Career Bullying

When the medically-qualified individual first started as a pre-registration house officer in the early 1980s in the United Kingdom, there was no such thing as mentorship support for the junior doctor. The reportee told us that he and his colleagues “just had to sink or swim”. Unfortunately, the house-physician in the job alongside the reportee was a type 1 diabetic and long hours without time for food played havoc with his insulin requirements. The reportee wrote that his diabetic colleague simply walked away from a rota in a major British hospital in 1984, where he was expected to work extended daytime hours and also one night in every three, going up to one night in every two when a colleague was away (so-called internal cover). The reportee remarked that the “callousness of the situation meant that he was allowed to give up practicing Medicine without any murmur of support”. The colleague was seen as being weak.

The reality of the British National Health Service (NHS) at that time was that the reportee was then expected to do two jobs, rather than one. From direct conversations, the reportee stated that he still remembered the Consultant in charge bringing him into an office to shout at him as patients had complained about undue waiting times to be seen by a doctor. The reportee stated that he remembered with visceral anxiety the tears rolling down his cheeks as the Consultant ranted that he would see to it that the reportee never worked again. From the diary of November 1984, the entry stated

Not a word of support, not a word of understanding for someone who was not only doing two extremely busy jobs, but who was working every other night without seemingly a moment to reflect alone. He was so angry that he left the room, leaving me a quivering wreck, while muttering that I wasn’t a real man.

The reportee spoke to us in a recent interview about the Consultant

His threats of stopping my career seemed very real, because in those days, we needed a reference to obtain the next job. There was no such thing as a comfortable 3 to 5 year rotation, nor the warm embrace of the local Deanery to monitor progress. I resolved to show him that he was wrong and worked twice as hard – even coming in to the wards on weekends off to ensure that I had everything to his liking.

The reportee recalled that senior house officer jobs were not much better. In one London Teaching Hospital, he reminisced that the long since deceased Consultant used to physically hit the Senior Registrar on a regular basis. The reportee remembered the Consultant “picking up the slightly built guy by his white coat lapels and shaking him”. The result was that such demonstrable cruelty was transmitted downwards to every member of the team. Thoughts raced around the reportee’s head that perhaps the first Consultant was right after all: “I wasn’t a real man”. The reportee developed panic attacks which became overwhelming and he left his post too. He thought that because his father was a doctor, he was not just left to become another casualty of a harsh medical system, but after some time off work where no mentorship support was offered, the reportee was given a quieter job which had become vacant, as someone else had moved on from the post. He resolved from that moment on that if he ever got into a position of influence, he would look after the interests of anyone and everyone on the team. He learnt by example not to make the mistakes of his seniors.

The lack of concern for his welfare at the time still haunts the reportee though, as he remembered one of the other Consultants on the firm crossing to the other side of the road, rather than entertaining a conversation with him when he greeted her several months later.

The world has changed: junior doctor hours are controlled and are reasonable, there is time for them to grow academically and mentally and there is a structure which hunts out the individual bully.^2^ Much has been learnt over four decades, but this does mean that bullying has become more covert than overt.

### Later Career Bullying

However, institutional bullying seems to be still alive and well, even in the more measured medical set-up that exists today. The reportee worked in London and in Cambridge in his junior doctor years, before doing research at a well-known London post-graduate institution, where he eventually was given a Clinical Senior Lecturer position.

He worked hard, won grants from the British Medical Research Council (MRC), the Wellcome Trust, the European Union, a variety of charities and Big Pharma over the years. It was a successful career - however, the reportee felt he never quite fitted in. He recalled at interview that he felt that there was a whispering campaign –

I was unmarried and the things I was rumoured to do in my private life shocked me, all the more because nobody ever asked what I got up to at a weekend, whereas it was the first question asked of my colleagues with 2.5 children when they walked through the door on a Monday morning.

Just past the millennium, the reportee recalled that he was passed over for promotion, when his colleagues with lesser academic achievements were elevated to the professoriat. He was told that his face did not fit and that he needed to do more to prove himself. On enquiring what he needed to do for promotion, he was informed that his research was not “germane to institutional themes”. On asking what these were, the answer came back from the Professor of Medicine “Whatever I decide”. The reportee recanted that he felt vulnerable and that he was none the wiser.

This came in the face of an administrator telling him that he was not allowed to supervise male medical students as he was seen to be a “risk”. The reportee remembers crying uncontrollably to that lady, a stalwart of the Anglican Church, who was reported to look horrified at his unmanly response and went about her business. The reportee complained to the institution’s human resources department through their bullying service, but nobody took the opportunity to do anything, apart from registering the incident itself.

The reportee resolved again to work twice as hard and to prove everyone wrong, even if all of which he was seen to be guilty was unfounded. He resolved to try to make a positive difference each day to those around him. However, the problem of large institutions was and still is that real bullies can hide behind due process. Nothing changed when he complained openly about discrimination. He felt that he was seen to be the problem, rather than someone who was bringing to light institutional inequalities and unfairnesses.

He realised this all the more having been interviewed for the role as Head of the University’s anti-bullying campaign. He was told that while he interviewed very well, it was obvious to the panel that he cared too much – they were interested in appointing a quieter candidate who would fit in: someone who would not change the status quo.

### The Effects of Bullying on Our Patients

Another area where medical practitioners have to be careful is in projecting our own biases on to patients. Our experience of life should not negatively affect those whom we have the privilege of treating, because this is also a form of bullying.^3^ This raises the question of how bullying influences the ethics of professional practice. A case in point where this was highlighted in the reportee’s mind, was a friend of his mother, who had extremely large breasts and who found difficulty in breathing at night as a consequence, in addition to having back pain and a resultant kyphosis. She sought an opinion on breast reduction, but was told by her doctor that she should “just get on with it”. The physician explained that she also had large breasts herself and would not consider complaining or burdening the NHS with “such trivia”. After 6 years of feeling constrained by what she considered physical dysmorphism, his mother’s friend was only taken seriously when she lost her balance, tottered over and broke her hip. A breast reduction resulted relatively swiftly. While this is an extreme example, unconscious bias and associated bullying raises its head daily, because most doctors prefer compliant patients, rather than thinking of a remodelled doctor-patient relationship, based on partnership.

## Discussion

Bullying may be overt and obvious, but in the current medical climate, the problem has become more subtle and is often exercised through institutional policy, rather than direct confrontation.^4^ The more measured current training of junior doctors allows the opportunity for overt individualised bullying to be highlighted and contained,^5^,^6^ but there is still a long way to go before a totally “mental health friendly” environment can be proclaimed for those training in and practicing medicine. Nevertheless, while overt individual bullying is generally a lesser and more limited aspect of human behaviour in today’s medical environment, the complexities of large institutions, whether they be academic or healthcare-related do not lend themselves to transparency or a real root-and-branch assessment of unconscious bias. The interrelationship between the morally-disengaged involvement of bullies in the workplace and the reaction of those in power is complex,^7^ but organisations should have zero tolerance for covert or overt bullying with confidential reporting systems to mitigate a continuing problem.^8^,^9^

Athena SWAN is a centralised reporting mechanism monitoring United Kingdom universities, aimed at promoting gender equality and equal opportunities in the workplace.^10^ We have found that Athena SWAN and other exercises which were designed to reward good institutional behaviour became a mechanism to tick off a series of boxes and hide bullying and innate prejudices under the carpet.^10^

In order to solve this problem, we think that those running Athena SWAN and other programmes such as Stonewall 100 (a mechanism to reward lesbian, gay, bisexual and transexual [LGBT] equality in the workplace)^11^,^12^ should spend greater time in ascertaining worm’s eye opinions of inequality (be it gender-based, ethnic minority-related, anti-Semitic, Islamophobic, LGBT-associated or disability-engendered), rather than accepting the top-down institutional approach, which is rarely based in reality.^13^ In a caring medical profession, there should be no room for intolerance, unconscious bias or bullying.
